# Two-stage Bayesian hierarchical modeling for blinded and unblinded safety monitoring in randomized clinical trials

**DOI:** 10.1186/s12874-020-01097-6

**Published:** 2020-08-17

**Authors:** Junhao Liu, Jo Wick, Renee’ H. Martin, Caitlyn Meinzer, Dooti Roy, Byron Gajewski

**Affiliations:** 1 Department of Biostatistics & Data Science, University of Kansas Medical Center, Mail Stop 1026, 3901 Rainbow Blvd, Kansas City, KS 66160 USA; 2 Novartis, East Hanover, NJ 07936 USA; 3 Department of Public Health Sciences, Medical University of South Carolina, Charleston, SC 29425 USA; 4 Boehringer Ingelheim, Ridgefield, CT 06877 USA

**Keywords:** Two-stage monitoring, Bayesian hierarchical method, Blinded and Unblinded safety data

## Abstract

**Background:**

Monitoring and reporting of drug safety during a clinical trial is essential to its success. More recent attention to drug safety has encouraged statistical methods development for monitoring and detecting potential safety signals. This paper investigates the potential impact of the process of the blinded investigator identifying a potential safety signal, which should be further investigated by the Data and Safety Monitoring Board with an unblinded safety data analysis.

**Methods:**

In this paper, two-stage Bayesian hierarchical models are proposed for safety signal detection following a pre-specified set of interim analyses that are applied to efficacy. At stage 1, a hierarchical blinded model uses blinded safety data to detect a potential safety signal and at stage 2, a hierarchical logistic model is applied to confirm the signal with unblinded safety data.

**Results:**

Any interim safety monitoring analysis is usually scheduled via negotiation between the trial sponsor and the Data and Safety Monitoring Board. The proposed safety monitoring process starts once 53 subjects have been enrolled into an eight-arm phase II clinical trial for the first interim analysis. Operating characteristics describing the performance of this proposed workflow are investigated using simulations based on the different scenarios.

**Conclusions:**

The two-stage Bayesian safety procedure in this paper provides a statistical view to monitor safety during the clinical trials. The proposed two-stage monitoring model has an excellent accuracy of detecting and flagging a potential safety signal at stage 1, and with the most important feature that further action at stage 2 could confirm the safety issue.

## Background

Interest in monitoring and reporting drug safety during the execution of a clinical trial and careful monitoring throughout the development of a drug from pre-clinical to post-marketing stages, has grown at a remarkable rate in the past decade. This attentiveness to drug safety has inspired statistical methods development for monitoring and detecting potential safety signals during trial execution. Proposed methods include Bayesian and frequentist models for blinded and unblinded safety monitoring for randomized clinical trials [1, 2].

Blinding is the process of concealing treatment-related information from the people involved in a clinical trial, such as the sponsors, participants, and researchers. Blinding preserves the integrity of the study by minimizing the impact on study findings of conscious or unconscious biases that might result from knowledge of treatment [3, 4]. The disclosure of treatment group assignment during the trial is called unblinding. For medical or safety reasons, unblinding a trial is sometimes necessary to protect study participants. The unblinding process is generally pre-specified and detailed in the study protocol [3–5].

Data and Safety Monitoring Boards (DSMBs) are independent committees responsible for regular monitoring and reporting of clinical trial safety data [6–8]. The Food and Drug Administration (FDA) requires the formation of a DSMB in all trials that assess new interventions [9, 10]. Furthermore, the FDA guidance of safety assessment for the Investigational New Drug (IND) safety reporting recommends that unblinding is allowed and needed to identify the important safety information for serious adverse events during an ongoing clinical trial [11]. “Flagging” is the notification process that identifies a potential safety concern in the novel treatment being tested in a clinical trial [3, 4]. DSMBs play a critical role in safety flagging; monitoring and reporting both the interests of trial participants, and the scientific integrity of clinical trials [5]. DSMBs regularly review blinded reports and listings of safety data to make determinations on whether the observed risk profile of the drug is different than expected. However, the investigator needs to be blinded to safety analyses throughout the conduction of the clinical trial. This paper investigates the potential impact of the process of the blinded investigator identifying a potential safety signal that the DSMB should further investigate with an unblinded safety data analysis.

The periodic safety reports reviewed by the investigator include a full listing of all adverse events (AE), as well as any serious adverse events (SAE) [12]. The report summarizes a trial’s clinical safety endpoints and AEs in terms of frequency of each event, the number of subjects having the event, severity of the event, and relatedness of the event to the study treatment. Because drug-related safety issues might occur at any time during the execution of a clinical trial, interim analyses of blinded safety data could help prevent such safety problems from escalating to significant concerns. Although blinded data analysis is less informative and does not provide a definitive treatment effect estimate, blinded safety data monitoring could identify potential safety issues ahead of scheduled DSMB meetings and prompt decisions regarding an unblinded analysis. For the purpose of accelerating the process of identifying important safety information, one feasible approach is to combine the blinded periodic safety monitoring with the intended unblinded data analysis. Additionally, the monitoring and evaluating of unblinded safety data will be performed based on the safety information from a blinded safety monitoring. Therefore, a two-stage monitoring method could be implemented to confirm and identify a safety signal for unblinded safety data at stage 2 once the potential AE(s) is flagged at stage 1.

Bayesian hierarchical approaches can be used for both blinded and unblinded data analysis by incorporating a prior safety profile of the control group or background rate of events and updating outcomes using accumulating data from the ongoing trial [13–15]. Prior assumptions on the safety profile must be made utilizing historical information or epidemiologic data. In this paper, a potential safety signal is identified by calculating the proportion of AEs from the pooled blinded safety data at stage 1. A blinded Bayesian hierarchical model based on Ball’s method of identifying possible safety signals is applied to the pooled blinded safety data [15]. Because of the Bayesian paradigm and its associated hierarchical models allow for automated adjustment for multiplicity and could reduce the family-wise error rate (FWER) in both stages, [16] a Bayesian hierarchical model that simultaneously models all AEs is considered [17–19]. A randomized clinical trial commonly refers to a control group and one or multiple active treatment groups with different dose levels. Therefore, at stage 2, a Bayesian hierarchical logistic model applied to unblinded data is used to simultaneously confirm whether the flagged safety signals are indeed safety issues [14, 15].

Throughout the trial, periodic blinded monitoring of events is conducted using Bayesian methods [16, 20]. Typically, investigators review blinded interim safety monitoring reports consisting of the proportion of subjects experiencing each AE with two-sided 95% credible intervals. If a possible safety signal is detected during blinded monitoring, a model-based estimate of the dose-response relationship on the relative risk will be provided to the DSMB [15].

This paper was originally motivated by the work of Ball and Wen who developed Bayesian objective early stopping rules for screening and monitoring safety in a randomized clinical trial using blinded treatment information [16, 20]. However, it is difficult for trial leadership to make decisions about stopping a trial only using blinded data. Therefore, this work proposes contributions to this area which include: 1) a potential Bayesian framework with a two-stage process for safety signal detection that facilitates decision-making using blinded data and then confirms it with the unblinded data; 2) and calculations of operating characteristics for this workflow. Most trials focus on power and sample size calculations (operating characteristics) for the primary efficacy analysis. This work extends this focus to safety by providing false positive and false negative rates for the proposed safety signal identification framework.

## Methods: two-stage Bayesian hierarchical models

### Stage 1: Bayesian blinded safety monitoring

The stage 1 Bayesian blinded statistical monitoring method assumes a randomized two-arm or multi-arm clinical trial. Subjects are continuously enrolled into the trial, and the first interim analysis occurs after a total *N* subjects have been enrolled into *I* + 1 arms, with *n*_0_ subjects enrolled into the control arm, and *n*_1_, *n*_2_, …, *n*_*I*_ subjects enrolled into the treatment arms.

#### Beta-binomial model

According to Wen and Ball’s Beta-Binomial model, [16, 20] the occurrence of the *j*^*th*^ AE among a total *J* types of AEs is denoted by *Y*_*ij*_ for the *i*^*th*^ dosage arm. In stage 1, *Y*_*j*_ is denoted as the total number of subjects experiencing the *j*^*th*^ AE reported in the pooled data, with the observed pooled incidence rate equal to ${\hat{\pi}}_j=\frac{Y_j\ }{N}$. Let ${\pi}_{M_j}$ represent the pre-specified expected pooled incidence rate across all dose levels of the *j*^*th*^ AE. The aggregated total across all arms (*Y*_*j*_) is assumed to have a Binomial distribution with occurrence probability *π*_*j*_ and *N*_*j*_ ≡ *N*. That is, ${Y}_j={\sum}_i{Y}_{ij}$ for *j* = 1, 2, …, *J* and *i* = 0, 1, 2, …, *I*, and the distribution of the *j*^*th*^ AE is given by,

$$ {Y}_j\sim Binomial\left({\pi}_j,N\right). $$

The occurrence probability *π*_*j*_ has a Beta prior distribution to facilitate a conjugate analysis. For example, assuming a *Beta*(1, 1) prior distribution for *π*_*j*_ results in a Beta posterior distribution,

$$ {\pi}_j\mid {Y}_j\sim Beta\left({Y}_j+1,N-{Y}_j+1\right). $$

The *j*^*th*^ AE may have a statistically significant safety signal if the posterior probability of its incidence rate being higher than the pre-specified expected pooled incidence rate exceeds a pre-specified critical value:

$$ P\left({\pi}_j>{\pi}_{M_j}| Blinded\ Data\right)>P\left( Critical\ Value\right). $$

#### Bayesian hierarchical blinded model

Considering the various types of AEs recorded in a clinical trial, multiplicity is a likely issue. Berry and Berry developed a Bayesian hierarchical model to handle multiple AEs simultaneously [17]. For the hierarchical model, it allows for the possible correlation between the AEs through the specified hyperparameters. Additionally, this approach allows for normal hierarchical models on the real line as opposed to the (0, 1) constraint, compared to the Beta-Binomial model. Therefore, the Beta-Binomial model and the Bayesian hierarchical model are combined to form the proposed Bayesian hierarchical blinded model.

For the hierarchical model, define *π*_*j*_ as a combination of control and treatment incidence rates, given by

$$ {\pi}_j={Q}_c\bullet {\pi}_{Ct{r}_j}+{Q}_T\bullet {\pi}_{Tr{t}_j}, $$

where *Q*_*c*_ is the proportionate sample size of the control arm, ${Q}_c=\frac{n_0}{N},$ and ${Q}_T=1-{Q}_c=\frac{\sum {n}_I}{N}$ is the proportionate sample size of the treatment arm(s); ${\pi}_{Ct{r}_j},{\pi}_{Tr{t}_j}$ are the incidence rates for the *j*^*th*^ AE in the control and treatment arms, respectively. Note that the ${\pi}_{Tr{t}_j}$ does not assume to be the same across treatment arms, it is a pooled incidence rate for *j*^*th*^ AE, and *Q*_*c*_ is usually fixed across different trial designs including designs that use response adaptive randomization. Assume that the incidence rate for the *j*^*th*^ AE in the control arm is equal to the expected pooled incidence rate, ${\pi}_{Ct{r}_j}\equiv {\pi}_{M_j}$. Then, the ${\pi}_{Tr{t}_j}$ across the treatment arms could be expressed by the difference between the pooled incidence rate *π*_*j*_ and expected pooled incidence rate ${\pi}_{M_j}$. Therefore, the logistic transformation is applied, yielding

$$ logit\left({\pi_{Trt}}_j\right)= logit\left({\pi}_{Ct{r}_j}\right)+{d}_j, $$

where *d*_*j*_ is the log-odds ratio of the probability of a safety event in the treatment relative to control for the *j*^*th*^ AE. The incidence rate of an AE is the same for control and treatment arms when *d*_*j*_ = 0. Priors are assigned to *d*_*j*_ using the following distribution:

$$ {d}_j\sim N\left({\mu}_d,{\sigma}_d^2\right). $$

The hyperparameters for the normal prior distribution of *d*_*j*_ have fixed distributions:

$$ {\mu}_d\sim N\left({\mu}_{d0},{\sigma}_{d0}^2\right) $$ and *σ*_*d*_~*Unif*(*U*_*a*_, *U*_*b*_),

where the hyperparameters ${\mu}_{d0},{\sigma}_{d0}^2,{U}_a,{U}_b$ are fixed constants. In general, due to the limited data, the prior information on *d*_*j*_ is typically lacking. However, the *d*_*j*_ is still identifiable for two reasons. The first is because the randomization allocation to the control arm is known and fixed as proportionate sample size (*Q*_*c*_, *Q*_*T*_) throughout the trial. The second is that the control arms rates priors are fixed at the expected incidence pooled rate. Therefore, in order to have a weakly informative impact on the prior distributions, and to carefully avoid overfitting or underfitting of the model, the weakly informative prior would be commonly recommended [21]. The specification of these hyperparameters depends on the application and is further discussed in the application section [22, 23].

Using the Bayesian hierarchical blinded model, posterior samples can be generated via Markov chain Monte Carlo (MCMC) methods, and the posterior probability *P*_*jS*1_ of a safety signal at stage 1 is given by ${P_j}_{S1}=P\left({\pi_{Trt}}_j>{\pi}_{M_j}| Blinded\ Data\right)$. After a specified number of subjects have been enrolled into the trial, during the interim safety analysis, the following decision rule can be applied for each AE to flag potential safety signals:

$$ {P_j}_{S1}\ge {P}_{crit_1}, $$

assuming some pre-specified critical value ${P}_{cri{t}_1}$. If the posterior probability exceeds the pre-specified critical value, an analysis of unblinded data can be performed to confirm the safety issue.

### Stage 2: Bayesian Unblinded safety monitoring

If at any point during stage 1 the blinded monitoring flags a safety signal, the unblinded dose-response effect for each AE will be modeled in stage 2 using a Bayesian hierarchical logistic model. It should be noted that only the AE(s) flagged at stage 1 will be unblinded and be subject to stage 2 monitoring. Under the scenario of various dose levels, assume the occurrence *Y*_*ij*_ of the *j*^*th*^ AE at the *i*^*th*^ dosage has a Binomial distribution with occurrence probability *π*_*ij*_. Assuming the number of subjects for the *i*^*th*^ dosage arm is represented by *n*_*i*_,

$$ {Y}_{ij}\sim Binomial\left({\pi}_{ij},{n}_i\right). $$

The logit function of *π*_*ij*_ is modeled with a linear predictor consisting of a fixed covariate effect of dose strength (*X*_*i*_):

*logit*(*π*_*ij*_) = *β*_0*j*_ + *β*_1*j*_*X*_*i*_, for *i* = 0, 1, 2, …, *I* and *j* = 1, 2, …, *J*.

In this model, the regression parameters *β*_0*j*_ and *β*_1*j*_ represent the control group parameters (intercept) and the regression parameters for the incremental effect of dose, respectively. Note that the logistic model could also be applied to a two-arm study. The hierarchical priors for *β*_0*j*_ and *β*_1*j*_ are given by

$$ logit\left({\beta}_{0j}\right)\sim N\left({\mu}_{\beta_0},{\sigma}_{\beta_0}^2\right);{\beta}_{1j}\sim N\left({\mu}_{\beta_1},{\sigma}_{\beta_1}^2\right), $$

where the parameter ${\mu}_{\beta_0}= logit\left({\pi}_{M_j}\right)$ allows for varying baseline incidence rates among the different types of AEs, and.

$$ {\mu}_{\beta_1}\sim N\left({\mu}_1,{\sigma}_1^2\right) $$ and $$ , {\sigma}_{\beta_0}\sim Unif\left({U}_1,{U}_2\right);{\sigma}_{\beta_1}\sim Unif\left({U}_3,{U}_4\right) $$.

The hyperparameters ${\mu}_1,{\sigma}_1^2$ and *U*_1_, *U*_2_, *U*_3_, *U*_4_ are fixed constants and are discussed in the application section.

The Bayesian hierarchical logistic model provides the posterior probability that the slope coefficient for dose is greater than 0; that is *β*_1*j*_ > 0. Slopes larger than 0 indicate a significantly increased occurrence probability of the *j*^*th*^ AE associated with the dose. The posterior probability *P*_*jS*2_ of a safety signal at stage 2 is given by

$$ {P_j}_{S2}=P\left({\beta}_{1j}>0| Unbl\mathrm{i} nded\ Data\right). $$

Therefore, *P*_*jS*2_ is compared to a pre-specified stage 2 critical value ${P}_{cri{t}_2}$, and a safety signal is confirmed when ${P_j}_{S2}\ge {P}_{cri{t}_2}$.

### Conduct of the trial

**Fig. 1:**
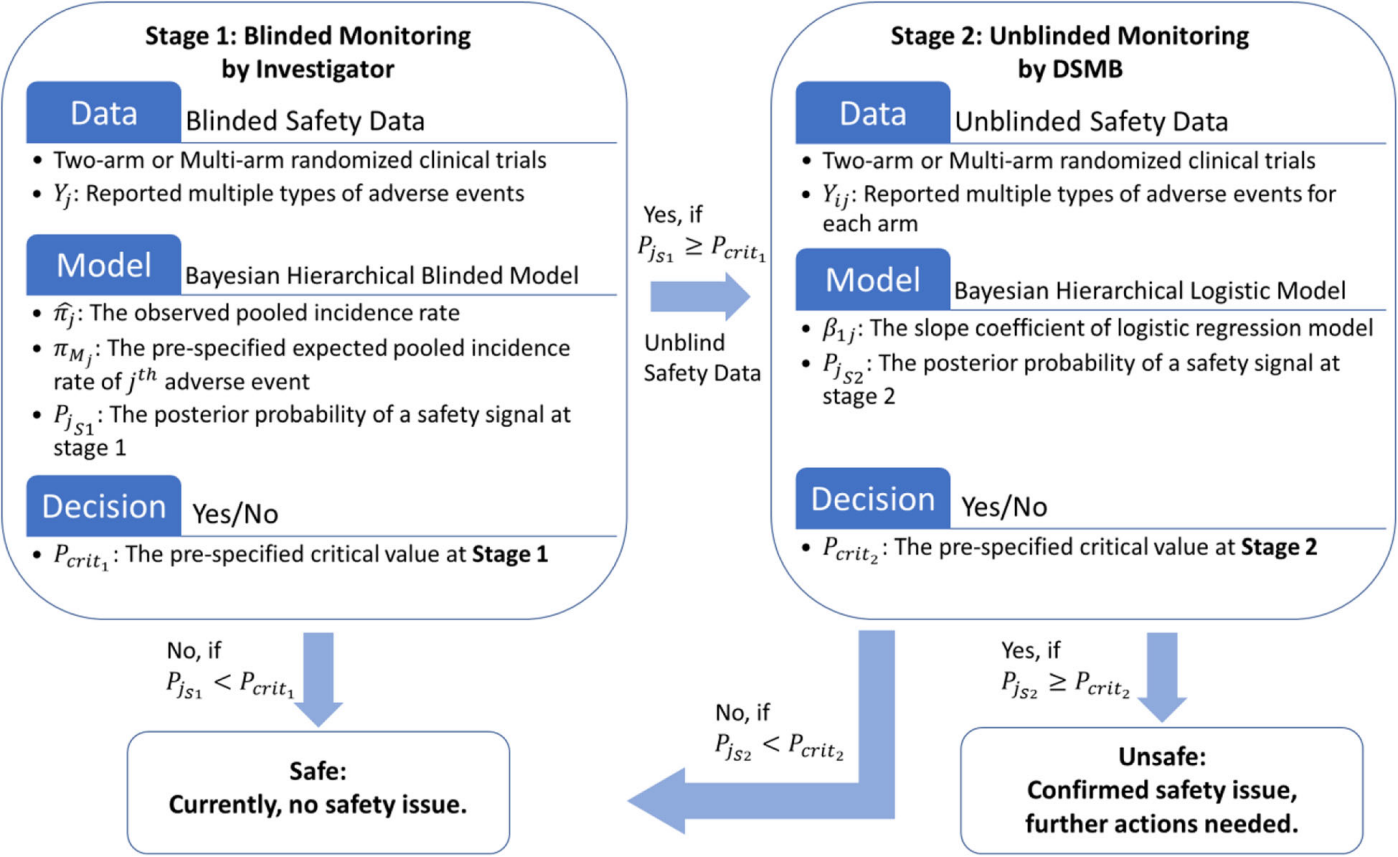
The flowchart of the Two-stage Bayesian safety monitoring for blinded and unblinded data. The figure displays the process of Two-stage Bayesian hierarchical monitoring, which starts with collecting the number of reported AE subjects in the pooled data, then goes through the Bayesian hierarchical blinded model to detect the potential safety signals at stage 1 for blinded safety data. Then, the Bayesian hierarchical logistic model is implemented to confirm the safety issue after the safety data being unblinded at stage 2

Given the models described in the previous section, we propose that a clinical trial be conducted via the sequential steps presented in Fig. 1.

Details are shown in the following steps:

1. Enrolled subjects are randomly assigned to each arm (either a simple two-arm trial or a multi-arm trial).
2. Interim safety analysis occurs after *N* subjects have been enrolled into *I* + 1 arms, with *n*_0_ subjects enrolled into the control arm and *n*_1_, *n*_2_, …, *n*_*I*_ subjects enrolled into treatment arms.
3. During stage 1, *Y*_*j*_ subjects report experiencing AE *j* at an interim point; that is, the observed pooled incidence rate for AE *j* is equal to ${\hat{\pi}}_j=\frac{Y_j\ }{N}$, and ${\pi}_{M_j}$ is the pre-specified expected pooled incidence rate of this AE.
4. Based on the Bayesian hierarchical blinded model, the posterior probability *P*_*jS*1_ of a safety signal at stage 1 is given by ${P_j}_{S1}=P\left({\pi_{Trt}}_j>{\pi}_{M_j}| Blinded\ Data\right)$. *P*_*jS*1_ is compared to the pre-specified critical value ${P}_{cri{t}_1}$. Once the model identifies a potential safety signal, ${P_j}_{S1}\ge {P}_{crit_1}$, the safety data for the *j*^*th*^ AE is unblinded and moved to stage 2.
5. During stage 2, only those AE(s) that have been flagged at stage 1 are examined. The Bayesian hierarchical logistic model provides the posterior probability of a safety signal *P*_*jS*2_ = *P*(*β*_1*j*_ > 0| *Unblinded Data*), which is compared to the pre-specified stage 2 critical value ${P}_{cri{t}_2}$. A safety issue is confirmed when ${P_j}_{S2}\ge {P}_{cri{t}_2}$.
6. Repeat at each interim point, updating ${\hat{\pi}}_j$ for stage 1. At any point a safety signal is detected, follow the decision rules above to confirm the potential safety issue.

## Case study

Consider a multi-arm case study of the Hyperbaric Oxygen Brain Injury Treatment (HOBIT) trial [24, 25]. HOBIT is a phase II clinical trial adaptive design for selecting the optimal dose regimen of hyperbaric oxygen (HBO) treatment, defined as the regimen (hyperbaric oxygen with or without normobaric oxygen at different pressure levels) which produces the greatest improvement in the rate of good neurological outcome versus standard care for subjects with severe traumatic brain injury.

For the HOBIT trial, the randomization occurs via the study-specific password-protected website accessed by an authorized research coordinator or investigator at the clinical site. Subjects are considered to be enrolled at the time of randomization, regardless of whether or not they start or complete study treatment. The trial uses the intent-to-treat randomized sample, where subjects are classified by the Oxygen Toxicity Units dose in which they are randomized, regardless of the dose received. The data for interim analysis (for efficacy) are collected from the subjects who have been randomized for more than 4 weeks from the time of the data freeze. In addition, the interim analysis of safety monitoring occurs after *N* = 53 subjects have enrolled into the trial. In this paper, the hypothetical scenarios of interim safety analysis occur after 53 subjects have enrolled into the trial, with 11 subjects enrolled into the control arm and 6 subjects enrolled for each treatment arm. However, this number changed to 56 with sample size modified to 9 for the “2.5 ATA + NBH” treatment arm, in the HOBIT trial. The comparison of AEs is between the control arm with seven treatment arms, where the sample size and dosage for eight arms are given in Table 1.

**Table 1:** The dosage and sample size for each dose-response arm within HOBIT trial

Eight Arms		Dose (Oxygen Toxicity Units)	Sample Size (*N* = 53)
1	Control (1.0 ATA)	0.0	11
2	1.5 ATA	2.60	6
3	2 ATA	4.17	6
4	NBH (100% FiO2 at 1.0 ATA)	5.40	6
5	2.5 ATA	5.92	6
6	1.5 ATA + NBH	6.20	6
7	2 ATA + NBH	7.76	6
8	2.5 ATA + NBH	9.52	6

### Adverse event of special interest

The review of safety data focuses on the following AEs potentially associated with hyperbaric oxygen treatment or in the transfer of subjects to getting their treatments. This subject population presents with significant morbidity with respect to all the below AEs; as such, it is important to evaluate the presence of events concerning temporal relationship to treatment (i.e., novel onset or worsening) as well as its relationship across doses. Therefore, the major individual AEs with clinical relevance and expected event rate are listed in Table 2. Additionally, the clinical information of each AE in Table 2 provides the simulation patterns from a modeling perspective.

**Table 2:** The most common AEs and the expected temporal and dose relationship

Adverse Event	Clinical Relevance	Expected Event Rate (*π*_*M*_)
Pneumothorax Induced by HBO therapy	**Abnormal collection of air in the pleural space between the lung and the chest wall, can result in steadily worsening oxygen supply.** This is a pressure related phenomenon that can also be caused by major trauma or medical procedure. As an AE it is expected to increase as a function of dose atmospheres, but not duration of exposure or number of days treatment. This is expected to occur during the dive and would result in aborting the treatment.	2%
Signs of Pulmonary Dysfunction	Signs of pulmonary dysfunction, including PaO2/FiO2 ≤ 200 or requiring PEEP > 10 cm of water to maintain a PaO2/FiO2 ratio of > 200. **This is an adverse event which may be related to total oxygen toxicity exposure and as such should increase with dose and number of treatments.** Symptoms are expected to progressively worsen over subsequent dives.	25%
Pneumonia	**This is an adverse event which is related to total oxygen toxicity exposure** and as such should increase with dose and number of treatments. Symptoms are expected to progressively worsen over subsequent dives.	40%
Critical decreased CPP (< 60 mmHg)	This AE is not specific to HBO therapy, but is associated with poor outcome (reperfusion). It is expected to be the same in all groups but could demonstrate differences if the process of transferring to the dive chamber causes increased AEs. **This should be analyzed as active vs. control.**	75%
Critical hypotension (MAP< 70 mmHg)	This AE is not specific to HBO therapy, but can be related to transfer from critical care unit (e.g. disconnecting and reconnecting of lines). It is expected to be the same in all groups but could demonstrate differences if the process of transferring to the dive chamber causes increased AEs. **This should be analyzed as active vs. control.**	75%
Seizures during HBO treatment	**These are expected to occur immediately proximal to treatment as a function of dose oxygen toxicity (rather than cumulative exposure).** It is possible to have multiple episodes of AE. Subjects with a baseline propensity to seize may elevate the numerator for this AE.	1%
Hypercarbia during transportation (PaCO2 > 45 mmHg)	This AE is not specific to HBO therapy, but related to transfer from critical care unit (e.g. disconnecting and reconnecting of lines). It is expected to be the same in all groups but could demonstrate differences if the process of transferring to the dive chamber causes increased AEs. **This should be analyzed as active vs. control.**	10%

All the AEs of special interest are summarized by preferred term and associated system-organ class according to the Medical Dictionary for Regulatory Activities (MedDRA) adverse reaction dictionary and by treatment group in terms of frequency of the event, number of subjects having the event, time relative to randomization, severity, and relatedness to the treatment. Cumulative incidences of the specific AE related to hyperbaric oxygen are compared across arms.

### Simulation study

In the simulation study, an example is provided by following the HOBIT trial design to demonstrate the two-stage safety monitoring process and decision criterion. As considered and discussed by Berry et al. and Gajewski et al. about the strategy to select the specification of the hyperparameters, the selection is determined by outcome type and expectation of the dose-response for the particular application [21, 24]. Therefore, in our application of the HOBIT trial, with the aim to minimize the informative impact on the prior distribution, and to avoid overfitting or overfitting for the model, [24] the hyperparameters described in Section 2 are assumed follow the fixed values: ${\mu}_{d0}=0,{\sigma}_{d0}^2={2}^2,{U}_a=0,{U}_b=3$ for the blinded model, and ${\mu}_1=0,{\sigma}_1^2={2}^2$ and *U*_1_ = 0, *U*_2_ = 3, *U*_3_ = 0, *U*_4_ = 3 for the unblinded model. Additionally, the *π*_*j*_ are defined as a combination of control incidence rate plus the all-treatment incidence rate, *π*_*j*_ = *Q*_*c*_ ∙ *π*_*Ctr*_ + *Q*_*T*_ ∙ *π*_*Trt*_, where *Q*_*c*_ = 0.2, *Q*_*T*_ = 0.8 were given by protocol information.

In order to understand the operating characteristics, several patterns of AEs are simulated. The simulation calculations for the two-stage Bayesian monitoring models were applied by MCMC methods, with the code presented in the Additional file 1. The results are based on 10,000 iterations of the study, each generated using 10,000 posterior samples after 1000 observations of burn-in.

### Two-stage Bayesian hierarchical safety monitoring models

Two approaches—a Beta-Binomial independent model and the hierarchical model—are applied to compare the family-wise error rate for blinded stage 1 safety data [26]. Table 3 provides the model comparisons for hypothetical observed event rates with the probability of flagged trials for the AE of special interest. Here, the *π*_0_ is the true incidence rate for the specific AE and does not assume to be the same for all non-control arms. For the case study at blinded stage, the simulated incidence rate were generated unequally under various scenarios. The choice of critical value should be pre-specified and depend on the severity of the AE and should be decided upon by investigators based on their experience. For the first interim analysis, a sample size *N* = 53 and a stage 1 critical value of 0.9 are assumed. For each specific AE, we assume the observed incidence rate varies under different scenarios, from the expected rate (safe rate) to a higher rate (unsafe rate). Based on the true incidence rate and the expected event rate, the proportion of flagged trials are given in Table 3.

**Table 3:** The model comparison for hypothetical observed event rates with probability of flagged trials for the AE of special interest between Beta-Binomial independent model and Bayesian hierarchical model

Example AEs	Expected Event Rate (*π*_*M*_)	True Proportion (*π*_0_)	Proportion of Flagged Trials After N = 53 Subjects Treated	
			Beta-Binomial Independent Model $\left({P}_{cri{t}_{Ind}}=0.9\right)$	Bayesian Blinded Hierarchical Model $\left({P}_{crit_I}=0.9\right)$
Pneumothorax Induced by HBO therapy	2%	2%	0.14	0.02
	2%	12%	1.00	0.98
	2%	17%	1.00	1.00
Signs of Pulmonary Dysfunction	25%	25%	0.10	0.05
	25%	35%	0.83	0.75
	25%	40%	0.97	0.91
Pneumonia	40%	40%	0.09	0.06
	40%	50%	0.76	0.72
	40%	55%	0.96	0.90
Critical decreased CPP (< 60 mmHg)	75%	75%	0.10	0.07
	75%	85%	0.89	0.87
	75%	90%	1.00	0.99
Critical hypotension (MAP< 70 mmHg)	75%	75%	0.10	0.06
	75%	85%	0.89	0.88
	75%	90%	1.00	0.99
Seizures during HBO treatment	1%	1%	0.27	0.01
	1%	11%	1.00	0.99
	1%	16%	1.00	1.00
Hypercarbia during transportation (PaCO2 > 45 mmHg)	10%	10%	0.12	0.04
	10%	20%	0.95	0.86
	10%	25%	1.00	0.97
	No Signal Pattern		Family-Wise Error Rate (FWER)	
Overall Adverse Events	Safe: *π*_0_ = *π*_*M*_		0.62	0.19

Table 3 shows that as the observed incidence rate increases, the proportion of flagged trials increases. For example, based on historical data the expected event rate of “Signs of Pulmonary Dysfunction” is 0.25 and the critical value at stage 1 is 0.9. Therefore, the Beta-Binomial independent model decision rule is

$$ P\left({\pi}_j>0.25| Blinded\ Data\right)\ge 0.9. $$

The Bayesian blinded hierarchical model decision rule is given by

$$ P\left({\pi_{Trt}}_j>0.25| Blinded\ Data\right)\ge 0.9. $$

In this case, a safety signal would be flagged if the posterior distribution provides evidence that the overall incidence rate likely exceeds 0.25. Additionally, under the scenario of no signal pattern, family-wise error rates are calculated across all seven AEs. The Bayesian hierarchical model is recommended for safety signal detection, since it accounts for multiplicities and it reduces the FWER because of the shrinkage at each AE type that is induced by the hyperparameters. The hierarchical model shows a smaller FWER compared to the Beta-Binomial independent model, as well as the smaller proportion of flagged trials.

Stage 2 includes all AEs that were flagged in stage 1. After unblinding the safety data, the dose-response effect of the AEs is modeled using Bayesian hierarchical logistic regression. The logit function of incidence rate for each arm was modeled using a linear predictor consisting of a fixed covariate effect of dose strength (*X*_*i*_) for each patient, where *X*_*i*_ is summarized as oxygen toxicity units/100 [24, 25].

**Fig. 2:**
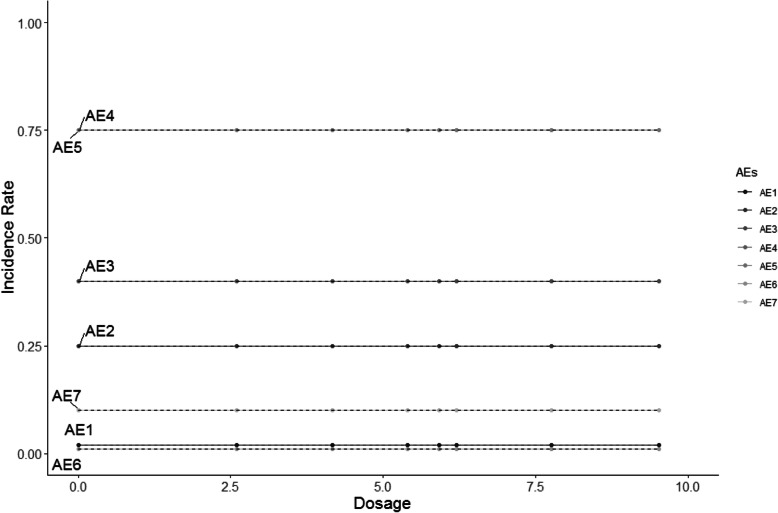
The scenario I of no effect across all seven AEs under various flat incidence rate as dosage increases for eight arms. (The dashed line is the expected incidence rate for each AE)

**Fig. 3:**
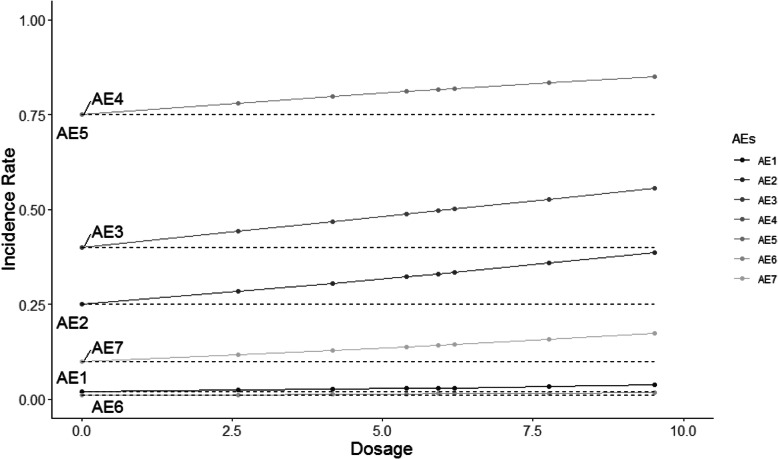
The scenario II of same effect across all seven AEs with safety issues under various increasing incidence rate as dosage increases for eight arms. (The dashed line is the expected incidence rate for each AE)

**Fig. 4:**
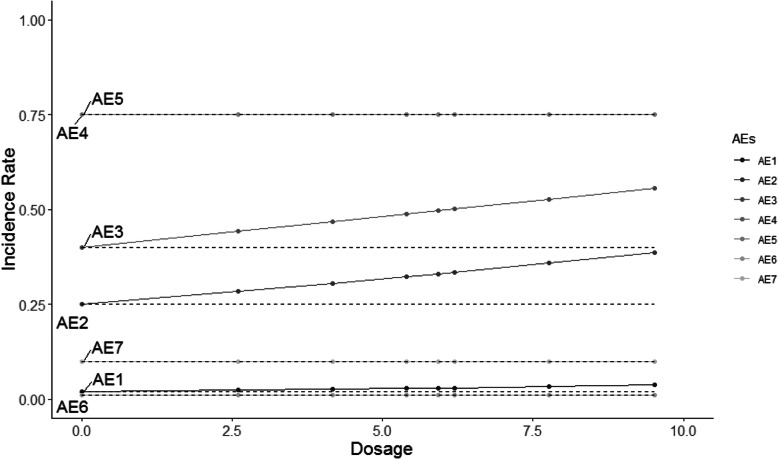
The scenario III of same effect for the first three AEs with safety issues (No effect for the rest) under various non-decreasing incidence rate as dosage increases for eight arms. (The dashed line is the expected incidence rate for each AE)

**Fig. 5:**
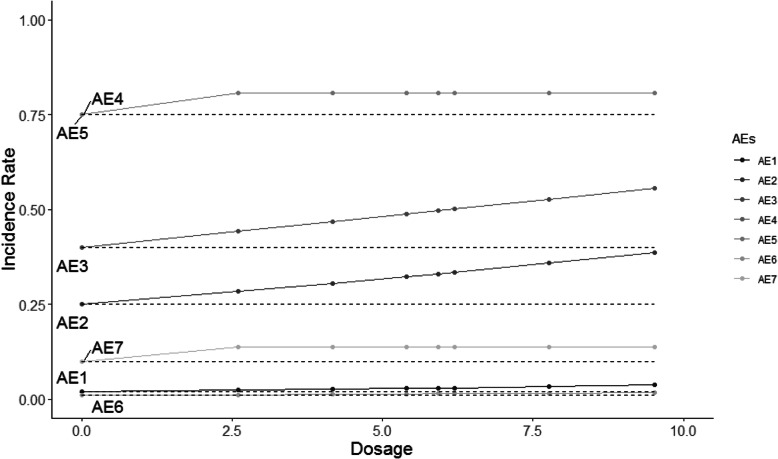
The scenario IV of three AEs with flat effect relationships and same effect for rest of AEs with safety issues under various non-decreasing incidence rate as dosage increases for eight arms. (The dashed line is the expected incidence rate for each AE)

**Fig. 6:**
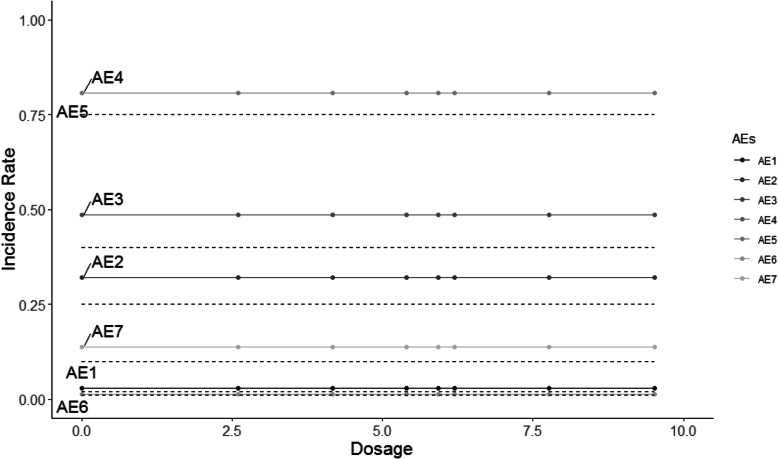
The scenario V of flat effect where both the control and treatment arms are the same but higher than the expected incident rate under various flat incidence rate as dosage increases for eight arms. (The dashed line is the expected incidence rate for each AE)

The HOBIT trial is an eight-arm trial, and non-decreasing incidence rates are assumed for each dose as dosage increases. Five different scenarios are considered and shown in Figs. 2, 3, 4, 5 and 6, where the average signal corresponds to the blinded scenarios. These figures show the simulation study patterns of various non-decreasing incidence rates as dosage increases for eight arms. In addition, the proposed two-stage models could be tested on the performance of detecting and confirming those safety issues under different AEs with varied expected incidence rates. For each scenario, the x-axis represents the dosage for each arm, and the y-axis indicates the observed incidence rate *π*_*j*_. A scenario of no effect across all AEs is considered (Fig. 2), and a scenario that assumes the same effect for all the AEs but with a safety issue (Fig. 3) is also considered. The same effect scenario was chosen to investigate the situation where the hierarchical model does very well. This assumption is relaxed in the next scenario. In another scenario, only the first three AEs (Pneumothorax Induced by HBO therapy, Signs of Pulmonary Dysfunction, and Pneumonia) have a safety issue (Fig. 4). Under this case, the proposed model is tested on a situation that only 3 AEs have a safety issue with no issue for the rest. In the HOBIT trial, as described in the Table 2, some AEs (Critical decreased CPP, Critical hypotension, and Hypercarbia during transportation) should be analyzed as active vs. control because they could potentially have a flat effect (e.g., in the logistic regression), thus these are modeled separately at stage 2 in scenario IV (Fig. 5). Additionally, a flat effect is considered where both the control and treatment rates are the same but higher compared to the expected incidence rate (Fig. 6). Under this case, assume the control group has a higher incidence rate than the expected, which is a safety issue. Then the proposed model is applied to test the detection and confirmation performance for this scenario.

## Results

The proposed safety monitoring process starts once 53 subjects have been enrolled into the trial for the first interim analysis. The Bayesian hierarchical blinded model is applied for detecting the potential safety signals at stage 1 and moves to stage 2 once the model detects a safety signal. In stage 2, the confirmation of safety is monitored using a Bayesian hierarchical logistic model. The critical value for stage 1 is set to 0.9 following the protocol and varied critical values for stage 2 from liberal to conservative. Three critical values situations are as follows: 1) Liberal: (0.9, 0.7), Medium: (0.9, 0.8), Conservative: (0.9, 0.9). Operating characteristics and FWER results are given in Table 4 for (A) no effect scenario I, Table 5 for the (B) same effect for all the AEs with safety issue scenario II, and Table 6 for the (C) same effect for three AEs with safety issue (No effect for the rest) scenario III, Table 7 for the (D) three AEs with flat effect relationships and same effect for the rest with safety issues scenario IV, and Table 8 for the (E) flat effect where both the control and treatment arms are the same but higher than the expected incidence rate scenario V.

**Table 4:** The probability of flagged trials for the AEs under the no effect for scenario I

Example AEs (True Proportion; Expected Event Rate)	Blinded: Proportion with signal for early termination Unblinded: Termination Proportion with confirmed by unblinded data	(*P*_*crit*1_, *P*_*crit*2_)		
		(0.9, 0.7)	(0.9, 0.8)	(0.9, 0.9)
Pneumothorax Induced by HBO therapy (*π* = 2%; *π*_*M*_ = 2%)	Blinded	0.02	0.02	0.01
	Unblinded	0.58	0.40	0.16
	**Overall Rate**	**0.01**	**0.01**	**0.00**
Signs of Pulmonary Dysfunction (*π* = 25%; *π*_*M*_ = 25%)	Blinded	0.05	0.05	0.06
	Unblinded	0.40	0.26	0.16
	**Overall Rate**	**0.02**	**0.01**	**0.01**
Pneumonia (*π* = 40%; *π*_*M*_ = 40%)	Blinded	0.06	0.06	0.06
	Unblinded	0.41	0.24	0.13
	**Overall Rate**	**0.03**	**0.02**	**0.01**
Critical decreased CPP (< 60 mmHg) (*π* = 75%; *π*_*M*_ = 75%)	Blinded	0.06	0.06	0.07
	Unblinded	0.45	0.30	0.16
	**Overall Rate**	**0.03**	**0.02**	**0.01**
Critical hypotension (MAP< 70 mmHg) (*π* = 75%; *π*_*M*_ = 75%)	Blinded	0.06	0.07	0.06
	Unblinded	0.44	0.31	0.16
	**Overall Rate**	**0.03**	**0.02**	**0.01**
Seizures during HBO treatment (*π* = 1%; *π*_*M*_ = 1%)	Blinded	0.01	0.01	0.01
	Unblinded	0.53	0.40	0.11
	**Overall Rate**	**0.00**	**0.00**	**0.00**
Hypercarbia during transportation (PaCO2 > 45 mmHg) (*π* = 10%; *π*_*M*_ = 10%)	Blinded	0.04	0.04	0.04
	Unblinded	0.41	0.27	0.14
	**Overall Rate**	**0.01**	**0.01**	**0.01**
		Family-Wise Error Rate (FWER)		
Overall Adverse Events	Blinded	0.18	0.17	0.19
	Unblinded	0.57	0.44	0.25
	**Overall Rate**	**0.10**	**0.08**	**0.05**

**Table 5:** The probability of flagged trials for the AEs under the same effect for all the AEs with safety issue for scenario II

Example AEs (True Proportion; Expected Event Rate)	Blinded: Proportion with signal for early termination Unblinded: Termination Proportion with confirmed by unblinded data	(*P*_*crit*1_, *P*_*crit*2_)		
		(0.9, 0.7)	(0.9, 0.8)	(0.9, 0.9)
Pneumothorax Induced by HBO therapy^a^ (*π* = 3%; *π*_*M*_ = 2%)	Blinded	0.28	0.27	0.28
	Unblinded	0.79	0.62	0.33
	**Overall Rate**	**0.23**	**0.17**	**0.09**
Signs of Pulmonary Dysfunction^a^ (*π* = 31%; *π*_*M*_ = 25%)	Blinded	0.55	0.54	0.54
	Unblinded	0.76	0.63	0.42
	**Overall Rate**	**0.42**	**0.34**	**0.23**
Pneumonia^a^ (*π* = 48%; *π*_*M*_ = 40%)	Blinded	0.58	0.59	0.59
	Unblinded	0.75	0.65	0.44
	**Overall Rate**	**0.44**	**0.39**	**0.26**
Critical decreased CPP^a^ (< 60 mmHg) (*π* = 80%; *π*_*M*_ = 75%)	Blinded	0.55	0.55	0.56
	Unblinded	0.76	0.60	0.39
	**Overall Rate**	**0.42**	**0.33**	**0.22**
Critical hypotension^a^ (MAP< 70 mmHg) (*π* = 80%; *π*_*M*_ = 75%)	Blinded	0.56	0.56	0.54
	Unblinded	0.77	0.61	0.39
	**Overall Rate**	**0.43**	**0.34**	**0.21**
Seizures during HBO treatment^a^ (*π* = 1%; *π*_*M*_ = 1%)	Blinded	0.23	0.23	0.23
	Unblinded	0.79	0.61	0.31
	**Overall Rate**	**0.19**	**0.14**	**0.07**
Hypercarbia during transportation^a^ (PaCO2 > 45 mmHg) (*π* = 13%; *π*_*M*_ = 10%)	Blinded	0.44	0.43	0.43
	Unblinded	0.77	0.62	0.40
	**Overall Rate**	**0.33**	**0.26**	**0.17**

(Assume the AE with ^a^ has a safety issue)

**Table 6:** The probability of flagged trials for the AEs under the same effect for three AEs with safety issue (No effect for the rest) for scenario III

Example AEs (True Proportion; Expected Event Rate)	Blinded: Proportion with signal for early termination Unblinded: Termination Proportion with confirmed by unblinded data	(*P*_*crit*1_, *P*_*crit*2_)		
		(0.9, 0.7)	(0.9, 0.8)	(0.9, 0.9)
Pneumothorax Induced by HBO therapy^a^ (*π* = 3%; *π*_*M*_ = 2%)	Blinded	0.10	0.10	0.11
	Unblinded	0.68	0.48	0.27
	**Overall Rate**	**0.07**	**0.05**	**0.03**
Signs of Pulmonary Dysfunction^a^ (*π* = 31%; *π*_*M*_ = 25%)	Blinded	0.36	0.36	0.36
	Unblinded	0.70	0.55	0.36
	**Overall Rate**	**0.25**	**0.20**	**0.13**
Pneumonia^a^ (*π* = 48%; *π*_*M*_ = 40%)	Blinded	0.41	0.40	0.41
	Unblinded	0.68	0.57	0.37
	**Overall Rate**	**0.28**	**0.23**	**0.15**
Critical decreased CPP (< 60 mmHg) (*π* = 75%; *π*_*M*_ = 75%)	Blinded	0.15	0.16	0.15
	Unblinded	0.49	0.34	0.17
	**Overall Rate**	**0.07**	**0.05**	**0.02**
Critical hypotension (MAP< 70 mmHg) (*π* = 75%; *π*_*M*_ = 75%)	Blinded	0.15	0.16	0.16
	Unblinded	0.48	0.31	0.16
	**Overall Rate**	**0.07**	**0.05**	**0.03**
Seizures during HBO treatment (*π* = 1%; *π*_*M*_ = 1%)	Blinded	0.05	0.05	0.05
	Unblinded	0.59	0.39	0.20
	**Overall Rate**	**0.03**	**0.02**	**0.01**
Hypercarbia during transportation (PaCO2 > 45 mmHg) (*π* = 10%; *π*_*M*_ = 10%)	Blinded	0.10	0.11	0.10
	Unblinded	0.47	0.36	0.15
	**Overall Rate**	**0.05**	**0.04**	**0.02**

(Assume the AE with ^a^ has a safety issue)

**Table 7:** The probability of flagged trials for the AEs under three AEs with flat effect relationships and same effect for the rest with safety issues for scenario IV

Example AEs (True Proportion; Expected Event Rate)	Blinded: Proportion with signal for early termination Unblinded: Termination Proportion with confirmed by unblinded data	(*P*_*crit*1_, *P*_*crit*2_)		
		(0.9, 0.7)	(0.9, 0.8)	(0.9, 0.9)
Pneumothorax Induced by HBO therapy^a^ (*π* = 3%; *π*_*M*_ = 2%)	Blinded	0.25	0.26	0.26
	Unblinded	0.74	0.57	0.28
	**Overall Rate**	**0.19**	**0.15**	**0.07**
Signs of Pulmonary Dysfunction^a^ (*π* = 31%; *π*_*M*_ = 25%)	Blinded	0.53	0.53	0.53
	Unblinded	0.74	0.60	0.39
	**Overall Rate**	**0.39**	**0.31**	**0.21**
Pneumonia^a^ (*π* = 48%; *π*_*M*_ = 40%)	Blinded	0.56	0.57	0.58
	Unblinded	0.75	0.61	0.41
	**Overall Rate**	**0.43**	**0.35**	**0.24**
Critical decreased CPP^a^ (< 60 mmHg) (*π* = 80%; *π*_*M*_ = 75%)	Blinded	0.50	0.49	0.51
	Unblinded	0.66	0.51	0.30
	**Overall Rate**	**0.33**	**0.25**	**0.15**
Critical hypotension^a^ (MAP< 70 mmHg) (*π* = 80%; *π*_*M*_ = 75%)	Blinded	0.49	0.50	0.51
	Unblinded	0.66	0.51	0.29
	**Overall Rate**	**0.32**	**0.26**	**0.15**
Seizures during HBO treatment^a^ (*π* = 1%; *π*_*M*_ = 1%)	Blinded	0.21	0.22	0.22
	Unblinded	0.74	0.56	0.28
	**Overall Rate**	**0.16**	**0.12**	**0.06**
Hypercarbia during transportation^a^ (PaCO2 > 45 mmHg) (*π* = 13%; *π*_*M*_ = 10%)	Blinded	0.39	0.39	0.38
	Unblinded	0.67	0.50	0.28
	**Overall Rate**	**0.26**	**0.19**	**0.11**

(Assume the AE with ^a^ has a safety issue)

**Table 8:** The probability of flagged trials for the AEs under flat effect where both the control and treatment arms are the same but higher than the expected incident rate for scenario V

Example AEs (True Proportion; Expected Event Rate)	Blinded: Proportion with signal for early termination Unblinded: Termination Proportion with confirmed by unblinded data	(*P*_*crit*1_, *P*_*crit*2_)		
		(0.9, 0.7)	(0.9, 0.8)	(0.9, 0.9)
Pneumothorax Induced by HBO therapy^a^ (*π* = 3%; *π*_*M*_ = 2%)	Blinded	0.32	0.33	0.33
	Unblinded	0.45	0.24	0.09
	**Overall Rate**	**0.15**	**0.08**	**0.03**
Signs of Pulmonary Dysfunction^a^ (*π* = 32%; *π*_*M*_ = 25%)	Blinded	0.60	0.60	0.59
	Unblinded	0.40	0.25	0.12
	**Overall Rate**	**0.24**	**0.15**	**0.07**
Pneumonia^a^ (*π* = 49%; *π*_*M*_ = 40%)	Blinded	0.65	0.66	0.64
	Unblinded	0.40	0.27	0.13
	**Overall Rate**	**0.26**	**0.18**	**0.08**
Critical decreased CPP^a^ (< 60 mmHg) (*π* = 81%; *π*_*M*_ = 75%)	Blinded	0.62	0.63	0.62
	Unblinded	0.42	0.28	0.13
	**Overall Rate**	**0.26**	**0.18**	**0.08**
Critical hypotension^a^ (MAP< 70 mmHg) (*π* = 81%; *π*_*M*_ = 75%)	Blinded	0.61	0.62	0.61
	Unblinded	0.42	0.28	0.12
	**Overall Rate**	**0.26**	**0.17**	**0.07**
Seizures during HBO treatment^a^ (*π* = 1%; *π*_*M*_ = 1%)	Blinded	0.28	0.28	0.28
	Unblinded	0.44	0.26	0.09
	**Overall Rate**	**0.12**	**0.07**	**0.02**
Hypercarbia during transportation^a^ (PaCO2 > 45 mmHg) (*π* = 14%; *π*_*M*_ = 10%)	Blinded	0.49	0.49	0.49
	Unblinded	0.41	0.27	0.11
	**Overall Rate**	**0.20**	**0.13**	**0.05**

(Assume the AE with ^a^ has a safety issue)

The summarized information of simulation scenarios and results comparison is given in Table 9. The scenario I can be treated as baseline proportions of no effect for all the AEs, then compared to scenario II, the proportions increase a lot as all the AEs have safety issues in scenario II. In scenario III, the first three AEs show higher proportions and the rest keep smaller proportions, since the first three AEs have safety issue in the scenario III. The difference between scenario II and scenario IV is that AE4, AE5, and AE7, these three AEs could be analyzing with active vs. control pattern, then we change their incidence rate as flat effect relationship. By comparing scenarios II and IV, the proportions of those flat effect relationship AEs decrease, and the rest AEs proportions are much similar. Based on the scenario II, scenario V is considered where all the AEs have a flat effect where both the control and novel therapies treatment are the same but higher compared to the expected incidence rate. The proportions indicate the safety issue, and one interesting finding is that the model flags potential signals at the blinded stage but not at the unblinded stage with fewer proportions comparing to other scenarios.

**Table 9:** The summary table of simulation scenarios and results comparison

Scenarios	AE #1	AE #2	AE #3	AE #4	AE #5	AE #6	AE #7
Scenario I (Safe: #1234567)	Safe	Safe	Safe	Safe	Safe	Safe	Safe
	Baseline proportions						
Scenario II (Unsafe: #1234567 same effect)	Unsafe	Unsafe	Unsafe	Unsafe	Unsafe	Unsafe	Unsafe
	Compare to Baseline proportions scenario I						
	Proportions increase a lot (All effect scenario)						
Scenario III (Safe: #4,5,6,7; Unsafe: #1,2,3 same effect)	Unsafe	Unsafe	Unsafe	Safe	Safe	Safe	Safe
	Compare to Baseline proportions scenario I						
	Higher proportions			Smaller proportions			
Scenario IV (Unsafe: #4,5,7 flat effect; Unsafe: #1,2,3,6 same effect)	Unsafe	Unsafe	Unsafe	Unsafe	Unsafe	Unsafe	Unsafe
	Comparing to scenario II						
	Similar proportions			Proportion decreases	Proportion decreases	Similar proportion	Proportion decreases
Scenario V (Unsafe: #1234567 flat effect; unsafe for control arms)	Unsafe	Unsafe	Unsafe	Unsafe	Unsafe	Unsafe	Unsafe
	The model flags potential signals at the blinded stage but not at the unblinded stage with fewer proportions comparing to other scenarios.						

At stage 1, by setting the pre-specific critical value to 0.9, the proportion of flagged trials is very similar within each AE; and at stage 2, as the critical value varies from 0.7 (liberal) to 0.9 (conservative), the proportion of flagged trials decreases. Therefore, the overall proportion is calculated by multiplying the proportions of both stage 1 and stage 2, and the overall proportion decreases as the critical value changes.

For the safety analysis, the critical values needs to balance the false flagged rate and false non-flagged rate. For example, scenarios I and II have proportions of flagged trials that are respectively equal to 0.05 and 0.75, under the pre-specific critical value of 0.9 for two-stage blinded and unblinded analyses. Similarly, scenarios III, IV and V have the proportions equal to 0.34, 0.66, 0.33 of flagged trials respectively with the same two-stage pre-specific critical values. In some instances, these operating characteristics may not change. However, in other instances, the proposed approach may change with the monitoring of efficacy. For example, if the treatment truly has no impact on efficacy (e.g. under the null hypothesis) there would be little impact on the first interim analysis. However, suppose scenario II is true but the drug has a true alternative hypothesis that has a probability of 0.3 of reaching the final success criteria. This would be the case where the DSMB would be hard-pressed to stop a promising treatment because of safety. In fact, the probability of both a safety signal and efficacy signal is 0.75 multiply by 0.3, which equals 0.225, clearly not a negligible amount. The good news is in the scenario I for safety the identification of a false flagged trial is 0.05 and under the null hypothesis for efficacy has a probability of 0.01 of reaching the final success criteria. The probability of both a safety signal and efficacy signal is 0.0005.

The results show that the Two-stage Bayesian safety monitoring model can detect and flag a potential safety signal, and with the most important feature that further action at stage 2 could confirm the safety issue. In addition, the family-wise error rate is also applied to the scenario I for no effect across all arms, [26] as shown in Table 4. The FWER is around 0.18 at the blinded stage 1 and decreases from 0.57 to 0.25 as the critical value increases at the unblinded stage 2, which the FWERs are acceptable under the current sample size scenario. The overall FWER across all seven AEs is relatively small, with only 5% incorrectly flagged for both critical values set to 0.9. That is, the two-stage model has an excellent accuracy of safety signal detection and confirmation.

## Discussion

Both sponsors and the DSMB often desire interim safety monitoring for clinical trials. In this paper, a two-stage Bayesian monitoring method is proposed to evaluate whether the posterior probability of a safety signal exceeds a pre-specified critical value. The proposed two-stage monitoring method not only combines the safety monitoring for blinded and unblinded data, but it also offers a comprehensive approach for detecting a potential safety issue of blinded data during stage 1 and performing an analysis of unblinded data at stage 2 to confirm the safety issue.

The Beta-Binomial model was originally proposed by Ball, and further development of the Binomial model was introduced in his recent safety monitoring paper as well [27]. Although, other available statistical methods have been developed and established for blinded safety monitoring [28, 29]. We adhere to the Binomial blinded safety monitoring model in this paper, since the follow-up period was fixed and the AEs were counted once during the study as indicated in the statistical analysis plan of the HOBIT trial. However, other developed methods, [28, 29] for example, the Poisson model account for exposure time is also feasible and practical for the two-stage Bayesian monitoring framework.

Direction for future development include the Poisson model framework, because of exposure-time is as critical as a number of events for drug safety monitoring. In recent research studies, the Poisson likelihood model was often used in blinded safety analysis, while considering the exposure time of AEs [28, 29]. Furthermore, it would easily allow combining multiple studies with different starting times during safety monitoring. Under the assumption that the AE for a given patient occurs independently and with a constant rate, a Poisson model could be applied to monitor safety signal. In addition, another development move from specifying a fixed expected pooled incidence rate ${\pi}_{M_j}$ for adverse events to using an informative prior instead. This allows a fully Bayesian treatment for two-stage safety monitoring. Moreover, regarding the criteria for the safety signal confirmation at stage 2, the incremental effect of dose for the current model, which is the slope, larger than 0 is the only indicator for detecting a significantly increased occurrence probability of the AE associated with the dose. One limitation that the toxicity probability at the highest dose, which is a sufficient indicator of safety signal confirmation criteria, but not considered in current model. Therefore, the toxicity probability of the highest dose could be included for the future development.

With respect to the generalizability of the proposed two-stage monitoring model, it could also provide support to cancer studies which have relatively small incidence rates for some AEs. Future work could add the evaluation of unblinded safety data conducted adjusting for relative baseline covariates, such as age at baseline or sex. The severity of an AE could also be built into the model. Finally, because the performance of such models depends on prior knowledge and researchers’ experience about AE incidence rates, the model could consider the selection of critical values and expected incidence rates for decision criterion as well. In the current study, the critical values for both stage 1 and stage 2 were set to 0.9 following the example study protocol, but future studies could relax this value. Another interesting extension in stage 2 is to modify the structure of the model, for example, either as random intercept/slope, or some other models, such as non-linear dose level model, Bayesian normal dynamic linear model (NDLM) and EMAX models [24].

## Conclusion

The Beta-Binomial model and Bayesian hierarchical blinded model are considered and compared in stage 1, and the Bayesian hierarchical model shows a lower family-wise error rate than the Beta-Binomial model, thus illustrating how failing to properly account for multiplicities can result in unreliable inference, while approximately preserving the probability of correctly detecting AE types with a safety signal. In the simulation study assuming no safety signals, the FWER—the probability of at least one safety signal among all AEs—was tightly controlled. Furthermore, in the presence of a safety signal for some or all AEs, the two-stage monitoring model successfully detected and confirmed those safety signals.

In the event of a significant safety signal, the blinded executive team can request to be unblinded to safety data only. If there is a significant trend but some arms appear to be safe, the DSMB and study team can discuss which arms to terminate. The interim monitoring and analysis of safety data could help prevent safety problems from turning to significant concerns in an ongoing clinical trial.

In summary, the decision to terminate a trial due to safety concerns is not a purely statistical one. This is one reason the DSMB is not comprised entirely of statisticians. The two-stage safety procedure in this paper provides a statistical view to monitor safety during the clinical trials, but never represents the medical and clinical decisions. More evaluation research and collaboration with clinicians and safety team are needed, in order to advance the safety detection and monitoring.

## Supplementary information

**Additional file 1.**

## Data Availability

The datasets during and/or analyzed during the current study available from the corresponding author on reasonable request.

## Abbreviations

DSMB: Data and Safety Monitoring Board
FDA: Food and Drug Administration
IND: Investigational New Drug
AE: Adverse Event
SAE: Serious Adverse Event
FWER: Family-wise Error Rate
MCMC: Markov chain Monte Carlo
HOBIT trial: Hyperbaric Oxygen Brain Injury Treatment trial
HBO: Hyperbaric Oxygen
MedDRA: Medical Dictionary for Regulatory Activities
NDLM: Normal Dynamic Linear Model
