# Correction to: Two-stage Bayesian hierarchical modeling for blinded and unblinded safety monitoring in randomized clinical trials

**DOI:** 10.1186/s12874-020-01114-8

**Published:** 2020-09-11

**Authors:** Junhao Liu, Jo Wick, Renee’ H. Martin, Caitlyn Meinzer, Dooti Roy, Byron Gajewski

**Affiliations:** 1 Department of Biostatistics & Data Science, University of Kansas Medical Center, Mail Stop 1026, 3901 Rainbow Blvd, Kansas City, KS 66160 USA; 2 Novartis, East Hanover, NJ 07936 USA; 3 Department of Public Health Sciences, Medical University of South Carolina, Charleston, SC 29425 USA; 4 Boehringer Ingelheim, Ridgefield, CT 06877 USA

**Correction to: BMC Med Res Methodol 20, 211 (2020)**

**https://doi.org/10.1186/s12874-020-01097-6**

Following publication of the original article [1], the authors would like to correct a sentence in the second paragraph under the heading Discussion.

The sentence currently reads:

The Beta-Binomial model was originally proposed by Ball, and further development of the Binomial model was introduced in his recent safety monitoring paper as well [27].

The sentence should read:

The Beta-Binomial model was originally proposed by Ball, and further development of bridging blinded and unblinded analysis was introduced in Lin’s recent safety monitoring paper as well [27].
