# Supplementary material for: Two-stage Bayesian hierarchical modeling for blinded and unblinded safety monitoring in randomized clinical trials
Source: BMC Med Res Methodol. 2020 Aug 17;20:211. doi: 10.1186/s12874-020-01097-6 (PMC7433072; doi:10.1186/s12874-020-01097-6)
Supplement: Supplementary file 1 — Additional file 1. [file 12874_2020_1097_MOESM1_ESM.docx]

**Appendix**

**BUGS Code:**

**Bayesian hierarchical blinded model:**

model

{

for( j in 1 : 7) {

Y[j] ~ dbin(p[j], n)

p[j] <- 0.2*p1[j] + 0.8*p2[j]

p1[j] <- pm[j]

logit(p2[j]) <- logit(p1[j])+d[j]

d[j] ~ dnorm(mud,invsigd2)

Prob[j] <- step(p2[j]-pm[j])

}

mud ~ dnorm(0,.25)

invsigd2 <- 1/(sigd*sigd)

sigd ~ dunif(0,3)

PP <- step(mud)

}

**Bayesian hierarchical logistic model:**

model

{

for (i in 1 : K) {

sy[i] ~ dbin(p[I[i], J[i]], n[i])

logit(p[I[i], J[i]]) <- beta0[J[i]] + beta1[J[i]]*X[I[i]]

}

for (k in 1 : JT) {

beta0[k] ~ dnorm(muB0[k], invsigd2B0)

beta1[k] ~ dnorm(muB1, invsigd2B1)

muB0[k] <- logit(pic[k])

}

invsigd2B0 <- 1/(sigdB0*sigdB0)

sigdB0 ~ dunif(0,3)

muB1 ~ dnorm(0,.25)

invsigd2B1 <- 1/(sigdB1*sigdB1)

sigdB1 ~ dunif(0,3)

for (k in 1 : JT) {

PPB1[k] <- step(beta1[k])

}

}
